# Synthesis and Antibacterial Activity of New Thiazolidine-2,4-dione-Based Chlorophenylthiosemicarbazone Hybrids

**DOI:** 10.3390/molecules23051023

**Published:** 2018-04-26

**Authors:** Nazar Trotsko, Urszula Kosikowska, Agata Paneth, Tomasz Plech, Anna Malm, Monika Wujec

**Affiliations:** 1 Department of Organic Chemistry, Faculty of Pharmacy with Medical Analytics Division, Medical University of Lublin, 4A Chodźki, Lublin 20-093, Poland; agatapaneth@umlub.pl (A.P.); monika.wujec@umlub.pl (M.W.); 2 Department of Pharmaceutical Microbiology with Laboratory for Microbiological Diagnostics, Faculty of Pharmacy with Medical Analytics Division, Medical University of Lublin, 1 Chodźki, Lublin 20-093, Poland; urszula.kosikowska@umlub.pl (U.K.); anna.malm@umlub.pl (A.M.); 3 Department of Pharmacology, Faculty of Health Sciences, Medical University of Lublin, 4A Chodźki, Lublin 20-093, Poland; tomasz.plech@umlub.pl

**Keywords:** thiazolidine-2,4-dione, chlorophenylthiosemicarbazones, antibacterial activity

## Abstract

Series of new thiazolidine-2,4-dione-based chlorophenylthiosemicarbazone hybrids (**17**–**40**) were synthesized by the reaction of condensation chlorophenylthiosemicarbazides with formylphenyl 2-(2,4-dioxothiazolidin-5-yl/ylidene)acetates. New compounds were tested on reference strains of Gram-positive and Gram-negative bacteria. The antibacterial activity of target compounds was determined by broth dilution method. Most active compounds possess minimum inhibitory concentration (MIC) = 3.91 mg/L. These compounds were non-toxic at concentrations close to their antibacterial effect. The antibacterial activity of some compounds was similar to or higher than the activity of used reference drugs such as oxacillin and cefuroxime. The structure–activity relationships (SARs) analysis collectively suggests that at least two different molecular mechanisms of their antibacterial activity should be expected.

## 1. Introduction

The appearance of drug-resistant strains of pathogenic microorganisms is a serious medical problem in worldwide. There is an urgent need to discover novel effective antimicrobial agents. Discovery of new compounds with antimicrobial activity or finding new mechanisms of action for known compounds is an important task of medicinal chemistry [1]. 

The 4-thiazolidinone derivatives and their narrow group—thiazolidine-2,4-diones (TZDs)—are the object of special scientific studies. This is confirmed by numerous reviews [2,3,4,5,6]. This attention is due not only to the wide possibilities for chemical modification of these derivatives, but also to a diverse spectrum of pharmacological properties and affinity for various biological targets. Consequently, TZD derivatives are the object of great interest as sources of novel drug candidates with anti-inflammatory [7,8], antimicrobial [9,10,11], antidiabetic [12,13], and anticancer [14,15,16] effects.

Design of new compounds based on preferential scaffolds is one of the favourable directions in drug discovery. 

As we previously reported, substances with an N-N-C(=S)-N structural fragment exhibit antimicrobial activity at low non-toxic concentrations. This activity is similar to or better than reference substances: cefuroxime, ampicillin, and vancomycin [17,18,19,20,21]. Combination of these two mentioned scaffolds in one molecule seems to be a promising “hybrid pharmacophore” approach to new antibacterial agents. 

In the present research, we used a fragment of 4-(chlorophenyl)thiosemicarbazone as a structural motif containing N-N-C(=S)-N. This fragment was connected with TZD derivatives in five positions of a heterocyclic ring, leading to new TZD-chlorophenylthiosemicarbazone hybrids. New compounds were studied as antibacterial agents.

## 2. Results and Discussion

### 2.1. Rationale

**Figure 1:**
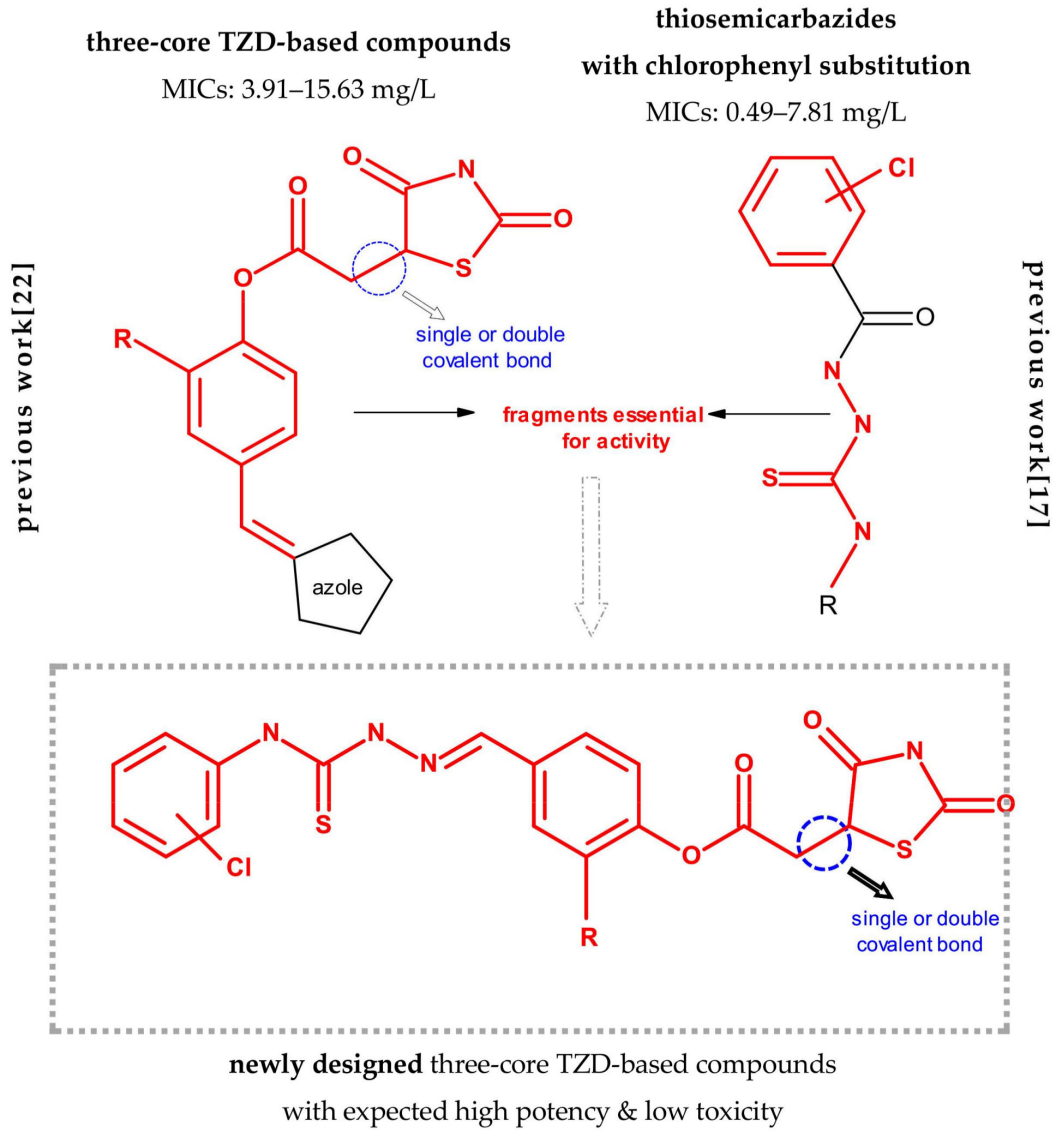
Design of novel three-core thiazolidine-2,4-diones (TZD)-based compounds with chlorophenyl substitution.

Our research group was focused on developing new antibacterial agents composed of three-core TZD-based scaffold (Figure 1). Previously reported compounds [22] were characterized by two nitrogenous five-membered heterocyclic rings connected by alkyl–phenyl–ester linker. The most potent of them had minimum inhibitory concentrations (MICs) against Gram-positive strains in the range of 3.91 mg/L to 15.63 mg/L, thereby indicating in some cases more effective action than those standard drug cefuroxime and ampicillin. Close inspection of the structure–activity relationships (SARs) revealed no direct effect of terminal TZD, rhodanine or 2-thiohydantoin core on the antibacterial response. Hence, we decided to modify initial structures by replacing the azole rings with the chlorophenylthiosemicarbazone fragment. Selection of such substituent was dictated by two factors; as we reported previously, compounds with N-N-C(=S)-N structural motif have the potential to display antibacterial activity at low, non-toxic concentration, similar to or even better than those for standard antibiotics cefuroxime, ampicillin or vancomycin [17,18,19,20,21]. Among them, the best antibacterial response was noted for those with the chlorophenyl substitution (Figure 1) [17]; the most active compounds had MICs as low as 0.49 mg/L (against *Micrococcus luteus* and *Bacillus subtilis*), making them promising starting structures for the development of new antibacterials. Thus, in this article, the active moieties for previously reported TZDs and those for thiosemicarbazides were combined together in one scaffold with the hope to obtain potent and non-toxic antibacterial agents (Figure 1). 

### 2.2. Chemistry

**Scheme 1:**
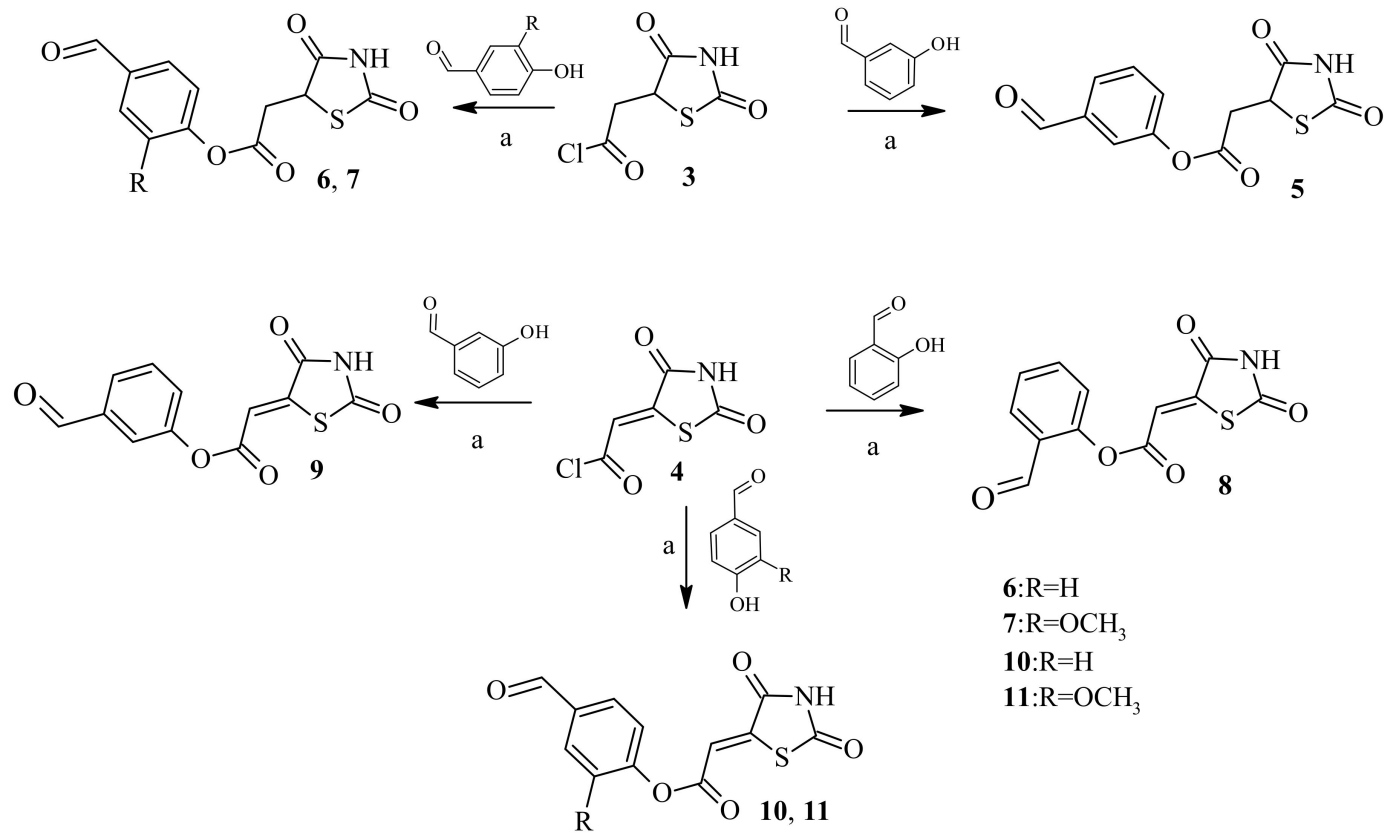
Synthesis of formylphenyl (2,4-dioxothiazolidin-5-yl/ylidene)acetates. Reagents and conditions: (a) pyridine, 1,4-dioxane, rt, after two hours acidified of solution of hydrochloric acid; yields 75–85%.

TZD-chlorophenylthiosemicarbazone hybrid compounds were obtained in a three-step procedure starting with appropriate (2,4-dioxothiazolidin-5-yl/ylidene)acetic acids (**1**, **2**). Compounds **1** and **2** were synthesized by the procedure described earlier [23]. The acids (**1**, **2**) by the reaction with thionyl chloride in anhydrous 1,4-dioxane medium were transformed into acid chlorides (**3**, **4**). Then (2,4-dioxothiazolidin-5-yl/ylidene)acetic acid chlorides (**3**, **4**) in the presence of pyridine were reacted with different hydroxybenzaldehydes (namely: salicylaldehyde, 3-hydroxybenzaldehyde, 4-hydroxybenzaldehyde and vanillin) to produce corresponding formylphenyl (2,4-dioxothiazolidin-5-yl/ylidene) acetates (**5**–**11**). The pathway of the reaction is illustrated in Scheme 1.

**Scheme 2:**
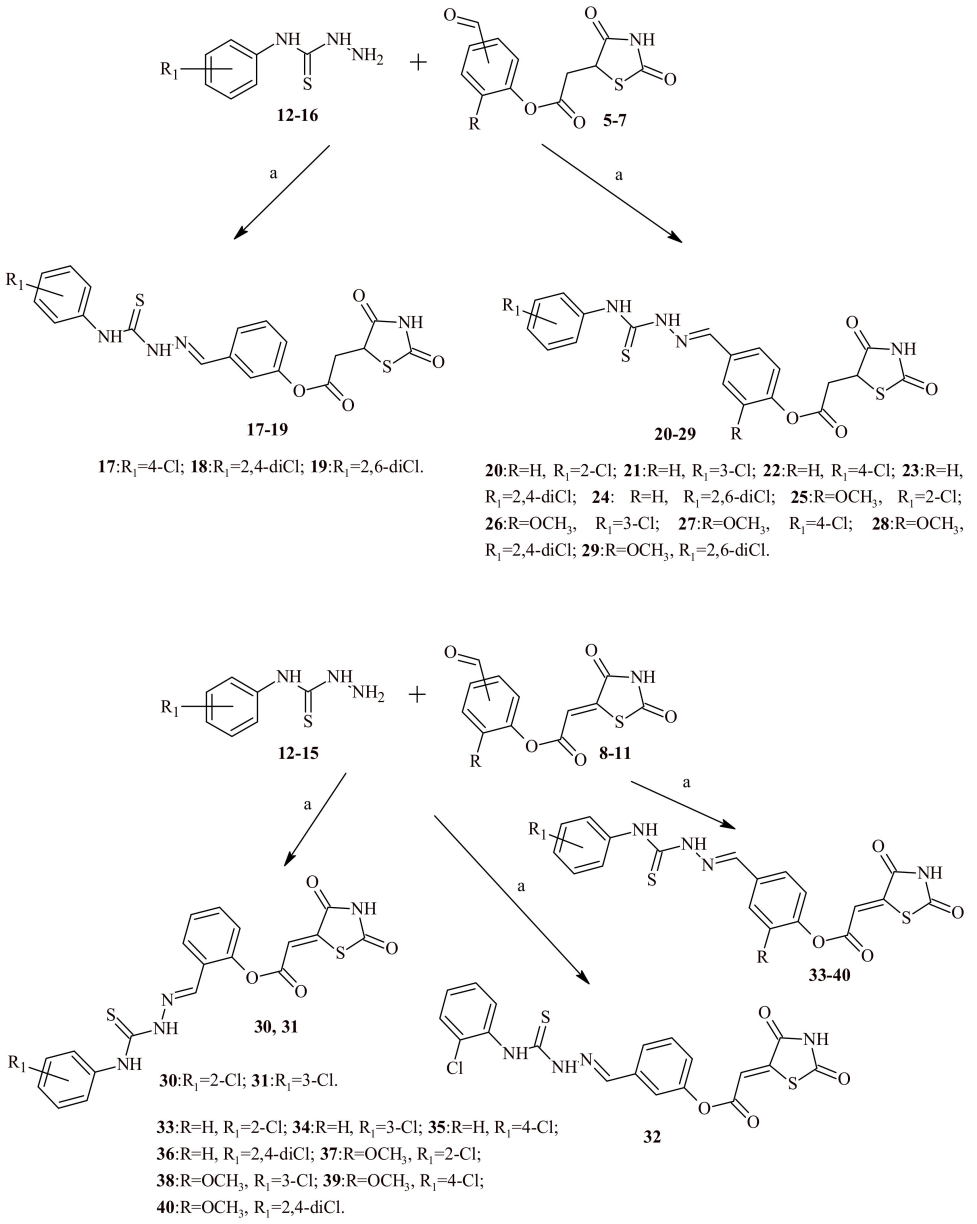
Synthesis of chlorophenylthiosemicarbazones (**17**–**40**). Reagent and conditions: (a) ethanol, reflux for 15 min; yields 62–92%.

The condensation of formylphenyl (2,4-dioxothiazolidin-5-yl/ylidene) acetates (**5**–**11**) with 4-(chlorophenyl)-3-thiosemicarbazides (**12**–**16**) in anhydrous ethanol medium in the presence of catalytic amounts of acetic acid led to obtaining corresponding chlorophenylthiosemicarbazones (**17**–**40**). The route of reaction is presented in Scheme 2.

The structure of target compounds (**17**–**40**) was confirmed by elemental analysis, ^1^H NMR and ^13^C NMR spectra. In ^1^H NMR spectra generally, the protons of CH=N group of all new synthesized compounds show singlet signal at δ ~ 8.15–8.31 ppm. The protons of NHCSNH group resonated as two singlets in the range of 10.08–10.23 ppm and 11.89–12.11 ppm correspondingly. The protons of NH group of thiazolidine ring appeared in the 12.12–12.19 ppm region for compounds (**17**–**29**) and the 12.91–13.04 ppm region for compounds as a singlet (**30**–**40**).

The presence of all carbon atoms for compounds (**17**–**40**) is confirmed by ^13^C NMR spectra. The signal of C=S group of thiosemicarbazone fragment appeared in the 176.3–177.8 ppm region. For the compounds **17**–**29**, which are derivatives of 2-(2,4-dioxothiazolidin-5-yl)acetic acid, carbon signal of two C=O group of thiazolidine ring appeared in the 166.4–172.7 ppm and 169.4–176.0 ppm regions. Signals of two C=O group of thiazolidine ring for the 2-(2,4-dioxothiazolidin-5-ylilidene)acetic acid derivatives **30**–**40** were visible at δ ~ 166.3–166.5 ppm and 169.2–169.4 ppm ranges correspondingly.

The detailed results of ^1^H NMR and ^13^C NMR spectra are presented in Section 3.

### 2.3. Antibacterial Screening

Initially, two series of TZD-chlorophenylthiosemicarbazones (**17**–**29** and **30**–**40**) were synthesized. The antibacterial results for MICs are reported in Table 1. The ciprofloxacin, cefuroxime and oxacillin were included as a control. The data from the assay showed that none of tested compounds had an inhibitory effect on the growth of Gram-negative bacteria examined up to a concentration of 1000 mg/L. In contrast, with the exception of **17**, **18**, **25**, **27**, **28**, **32**, **37**, all tested compounds possessed good to limited antibacterial activity. Among them, the best antibacterial response was observed for **38**; followed by **20**, **23**; moderately active **21**, **26**, **33**, **34**; and almost inactive **35**, **39** and **40**. From these results, together with those for three derivatives of the most potent **38**—compounds **41** (4-[(2,5-dioxoimidazolidin-4-ylidene)methyl]-2-methoxyphenyl (2,4-dioxo-1,3-thiazolidin-5-ylidene)acetate), **42** (4-[(2,5-dioxoimidazolidin-4-ylidene)methyl]-2-methoxyphenyl (2,4-dioxo-1,3-thiazolidin-5-yl)acetate) and **43** (2-ethoxy-4-[(5-oxo-2-thioxoimidazolidin-4-ylidene)methyl]phenyl (2,4-dioxo-1,3-thiazolidin-5-ylidene)acetate)—the following comments can be made: (i) since tested compounds varied widely in antibacterial activity, the chlorophenylthiosemicarbazone segment is not an optimized moiety for their activity; (ii) SARs are rather complex and cannot be easily explained. For example, compound **38** was a potent antibacterial agent, with even more effective action than those standard drug cefuroxime against B. subtilis and Bacillus cereus. The replacement of the chlorophenylthiosemicarbazone core with the hydantoin **41** abolished activity. In turn, the reduction of double bond in **41** furnished compound **42** with weak inhibitory activity against all bacterial strains while increasing the carbon chain from methyl to ethyl, providing compound **43** with mild activity. Further, slight change in geometry and electronic properties of **38** (**38 → 37**) also abolished activity, while the reduction of **37** (**37 → 25**) does not result in an active compound as was observed for **41** → **42**.

With this information in hand, we started exploring the proper substitution pattern on both terminal and central phenyl rings by initially adding the chloro substituent to potent antibacterial **20**. The chloro substitution at ortho position provided compound **24** with significantly lower activity; with the exception of *S. epidermidis*, all Gram-positive bacterial strains were able to grow at much higher concentrations as compared to **20** or were even almost insensitive. Low or even a lack of antibacterial activity was also noted for its structural isomers **19** and **18**, respectively. Important to note, compounds **18**, **19** and **24** with negligible activity are all structural isomers of **23**; one of the most potent among all tested compounds. In turn, substitution of weakly active **24** with the methoxy group afforded compound **29** with quite potent activity against *M. luteus* and *B. cereus*, whereas such modification in **33** (**33** → **37**) abolished activity. Finally, the effect of isomerization at the central phenyl ring was tested. For this purpose, compounds **17**, **30**, **31** and **32** were synthesized. Consistent with previous studies, compounds with meta substitution **17** and **32** were inactive. Compounds with ortho substitution **30** and **31**, however, showed an activity similar to or even better against *S. aureus* than the most potent antibacterial **38**, which in turn is in line with the trend in activity observed previously. 

Summing up, the SARs analysis collectively suggest that at least two different molecular mechanisms of antibacterial activity for TZD-based hybrid compounds are expected. Such observation, however, is not surprising: a similar trend was observed previously for thiosemicarbazide derivatives; one mechanism for their activity was associated with inhibition of bacterial topoisomerases, while the nature of the other is still unrecognized [18,19,21,24,25].

### 2.4. Bactericidal and Bacteriostatic Activity

Antibacterial studies were completed by the evaluation of MBCs (minimum bactericidal concentrations) that allowed to determine the bactericidal or bacteriostatic mechanism of action. On the basis of the MBC/MIC ratio, all compounds were mainly bacteriostatic in effect (MBC/MIC ≥ 8), with the exception of potent **20** that exhibited bactericidal activity (MBC/MIC ≤ 4) to half of the Gram-positive bacterial strains tested, further highlighting the concept of at least two molecular targets for antibacterial activity of title compounds.

### 2.5. Computational Studies

Finally, efforts were undertaken to correlate antibacterial activity with physicochemical properties, such as geometry of molecule, lipophilicity, refractivity, polarizability, surface area, volume, dipole moment, hardness, Mulliken electronegativity, total energy, E_HOMO_, E_LUMO_, HOMO*–*LUMO gaps, ESP charges, core–core interaction, heat of formation, electrostatic potential surfaces, electronic distribution of the frontier orbitals HOMO and LUMO orbitals. As we assumed, analysis of the SARs did not provide valuable information about the observed trend in bioactivity; the results showed that varying the substitution pattern of the chloro subunit or desaturation process leads to relatively small changes in geometry or physicochemical characterization but causes significant changes in antibacterial response (data not shown).

### 2.6. Cytotoxicity Studies

After confirming compounds **20**, **23**, **30**, **31** and **38** as potent antibacterial agents, we moved to examine their toxicity profile against human embryonic kidney cells (HEK-293). Compound **20** exhibited the best toxicity profile as it was non-toxic to HEK-293 cells to a concentration of 30.82 mg/L (Table 2); this represents an eight-fold difference between the MICs and the concentration where its significant toxicity is observed. 

Cytotoxic effect of the compounds was measured using MTT assay. Data are expressed as mean ± SD from three independent experiments. Summing up, all compounds were non-toxic at concentrations close to their antibacterial effect.

## 3. Materials and Methods 

### 3.1. Chemistry

Melting points were determined by using Fisher–Johns apparatus (Fisher Scientific, Schwerte, Germany) and are uncorrected. The purity of the compounds was checked by TLC on plates with silica gel Si 60 F_254_, produced by Merck Co. (Darmstadt, Germany). The ^1^H NMR and ^13^C NMR spectra were recorded by a Bruker Avance 300 MHz instrument using DMSO-d_6_ as solvent and TMS as an internal standard. Chemical shifts were expressed as δ (ppm). Elemental analyses were performed by AMZ 851 CHX analyser and the results were within ±0.4% of the theoretical value.

#### 3.1.1. Synthesis of Chlorophenylthiosemicarbazones (**17**–**40**)

To the mixture of 0.001 mol formylphenyl (2,4-dioxothiazolidin-5-yl/ylidene)acetate (**5**–**11**) and 0.001 mol of the appropriate 4-(chlorophenyl)-thiosemicarbazides (**12**–**16**) was added 5 mL of ethanol. The reaction mixture was heated under reflux for 15 min. After cooling, the precipitate was filtered off and washed with ethanol. After drying, precipitate was recrystallized from appropriate solvents. The compounds (**17**–**24**, **26**, **27**, **30**, **31**, **33**, **35**–**40**) were crystallized from butanol, compounds **25**, **29**, from propanol; compounds **28**, **34**, from acetic acid; and compound **32**, from mixture of DMF:H_2_O (2:1).

3-[{2-[(4-Chlorophenyl)carbamothioyl]hydrazinylidene}methyl]phenyl (2,4-dioxo-1,3-thiazolidin-5-yl)acetate (**17**). 

Yield 80%, mp 210–212 °C. ^1^H NMR (DMSO-d_6_) δ (ppm): 3.42–3.45 m (2H, CH-C**H**_2_); 4.85 dd (1H, C**H**-CH_2_, *J* = 6.8, 5.2 Hz); 7.05 s, 7.28–7.31 m, 7.41–7.61 m, 7.73–7.77 m, 7.92 t (8H, 3-O-C_6_H_4_ and 4-Cl-C_6_H_4_, *J* = 1.8 Hz); 8.16 s (1H, CH=N); 10.20 s, 11.97 s (2H, NHCSNH); 12.16 s (1H, NH, thiazolidine). ^13^C NMR (DMSO-d_6_) δ (ppm): 36.4; 46.8; 116.3; 120.1; 123.7; 128.3; 128.4; 130.0; 130.5; 136.3; 138.5; 142.5; 151.0; 169.6; 172.7; 175.9; 176.7. Anal. calc. for C_19_H_15_ClN_4_O_4_S_2_ (%): C 49.30; H 3.27; N 12.10. Found: C 49.17; H 3.19; N 12.09.

3-[{2-[(2,4-Dichlorophenyl)carbamothioyl]hydrazinylidene}methyl]phenyl (2,4-dioxo-1,3-thiazolidin-5-yl)acetate (**18**)

Yield 83%, mp 224–225 °C. ^1^H NMR (DMSO-d_6_) δ (ppm): 3.42–3.44 m (2H, CH-C**H**_2_); 4.85 dd (1H, C**H**-CH_2_, *J* = 6.8, 5.3 Hz); 7.06 s, 7.27–7.30 m, 7.45–7.62 m, 7.71–7.73 m, 7.90 t (7H, 3-O-C_6_H_4_ and 2,4-diCl-C_6_H_3_, *J* = 1.8 Hz); 8.17 s (1H, CH=N); 10.14 s, 12.00 s (2H, NHCSNH); 12.12 s (1H, NH, thiazolidine). ^13^C NMR (DMSO-d_6_) δ (ppm): 36.3; 46.6; 116.3; 120.0; 123.8; 127.9; 129.3; 130.5; 132.1; 132.4; 133.1; 136.3; 136.5; 142.4; 150.9; 164.4; 166.8; 169.5; 177.6. Anal. calc. for C_19_H_14_Cl_2_N_4_O_4_S_2_ (%): C 45.88; H 2.84; N 11.26. Found: C 45.83; H 2.85; N 11.18.

3-[{2-[(2,6-Dichlorophenyl)carbamothioyl]hydrazinylidene}methyl]phenyl (2,4-dioxo-1,3-thiazolidin-5-yl)acetate (**19**)

Yield 81%, mp 203–204 °C. ^1^H NMR (DMSO-d_6_) δ (ppm): 3.41–3.44 m (2H, CH-C**H**_2_); 4.85 dd (1H, C**H**-CH_2_, *J* = 6.7, 5.4 Hz); 7.06 s, 7.28–7.31 m, 7.35–7.41 m, 7.49–7.57 m, 7.72 d, 7.94 t (7H, 3-O-C_6_H_4_ and 2,6-diCl-C_6_H_3_, *J* = 7.5, 1.8 Hz); 8.16 s (1H, CH=N); 10.16 s, 12.08 s (2H, NHCSNH); 12.10 s (1H, NH, thiazolidine). ^13^C NMR (DMSO-d_6_) δ (ppm): 35.2; 46.8; 116.4; 119.8; 123.7; 128.8; 129.9; 130.5; 135.7; 136.4; 142.1; 145.7; 150.8; 164.4; 166.6; 169.4; 177.8. Anal. calc. for C_19_H_14_Cl_2_N_4_O_4_S_2_ (%): C 45.88; H 2.84; N 11.26. Found: C 45.77; H 2.80; N 11.29. 

4-[{2-[(2-Chlorophenyl)carbamothioyl]hydrazinylidene}methyl]phenyl (2,4-dioxo-1,3-thiazolidin-5-yl)acetate (**20**)

Yield 82%, mp 190–192 °C. ^1^H NMR (DMSO-d_6_) δ (ppm): 3.42–3.45 m (2H, CH-C**H**_2_); 4.86 dd (1H, C**H**-CH_2_, *J* = 7.0, 5.2 Hz); 7.21 d, 7.96 d (4H, 4-O-C_6_H_4_, *J* = 8.7 Hz); 7.32 td, 7.39 td, 7.56 dd 7.67 dd (4H, 2-Cl-C_6_H_4_, *J* = 7.8, 1.6 Hz); 8.16 s (1H, CH=N); 10.13 s, 12.02 s (2H, NHCSNH); 12.17 s (1H, NH, thiazolidine). ^13^C NMR (DMSO-d_6_) δ (ppm): 36.4; 46.8; 122.5; 127.7; 128.4; 129.3; 129.8; 130.6; 131.4; 132.5; 137.1; 142.4; 151.8; 169.5; 172.7; 176.0; 177.3. Anal. calc. for C_19_H_15_ClN_4_O_4_S_2_ (%): C 49.30; H 3.27; N 12.10. Found: C 48.98; H 3.19; N 12.11.

4-[{2-[(3-Chlorophenyl)carbamothioyl]hydrazinylidene}methyl]phenyl (2,4-dioxo-1,3-thiazolidin-5-yl)acetate (**21**)

Yield 83%, mp 193–194 °C. ^1^H NMR (DMSO-d_6_) δ (ppm): 3.42–3.45 m (2H, CH-C**H**_2_); 4.86 dd (1H, C**H**-CH_2_, *J* = 6.8, 5.3 Hz); 7.21 d, 8.01 d (4H, 4-O-C_6_H_4_, *J* = 8.7 Hz); 7.25–7.43 m, 7.57–7.63 m, 7.74–7.77 m (4H, 3-Cl-C_6_H_4_); 8.17 s (1H, CH=N); 10.20 s, 11.98 s (2H, NHCSNH); 12.16 s (1H, NH, thiazolidine). ^13^C NMR (DMSO-d_6_) δ (ppm): 36.5; 46.8; 122.4; 124.8; 125.5; 125.8; 129.5; 130.1; 132.4; 132.6; 141.0; 142.8; 151.8; 169.4; 172.7; 176.0; 176.3. Anal. calc. for C_19_H_15_ClN_4_O_4_S_2_ (%): C 49.30; H 3.27; N 12.10. Found: C 49.03; H 3.21; N 12.09.

4-[{2-[(4-Chlorophenyl)carbamothioyl]hydrazinylidene}methyl]phenyl (2,4-dioxo-1,3-thiazolidin-5-yl)acetate (**22**)

Yield 85%, mp 186–188 °C. ^1^H NMR (DMSO-d_6_) δ (ppm): 3.42–3.45 m (2H, CH-C**H**_2_); 4.86 dd (1H, C**H**-CH_2_, *J* = 7.0, 5.2 Hz); 7.21 d, 7.98 d (4H, 4-O-C_6_H_4_, *J* = 8.7 Hz); 7.43 d, 7.61 d (4H, 4-Cl-C_6_H_4_, *J* = 8.8 Hz); 8.16 s (1H, CH=N); 10.17 s, 11.94 s (2H, NHCSNH); 12.15 s (1H, NH, thiazolidine). ^13^C NMR (DMSO-d_6_) δ (ppm): 36.5; 46.9; 122.5; 128.1; 128.4; 129.4; 129.9; 132.5; 138.6; 142.6; 151.8; 169.5; 172.7; 176.0; 176.6. Anal. calc. for C_19_H_15_ClN_4_O_4_S_2_ (%): C 49.30; H 3.27; N 12.10. Found: C 49.35; H 3.25; N 12.00.

4-[{2-[(2,4-Dichlorophenyl)carbamothioyl]hydrazinylidene}methyl]phenyl (2,4-dioxo-1,3-thiazolidin-5-yl)acetate (**23**)

Yield 83%, mp 200–202 °C. ^1^H NMR (DMSO-d_6_) δ (ppm): 3.42–3.45 m (2H, CH-C**H**_2_); 4.86 dd (1H, C**H**-CH_2_, *J* = 7.0, 5.2 Hz); 7.21 d, 7.95 d (4H, 4-O-C_6_H_4_, *J*=8.7 Hz); 7.47 dd, 7.67 d, 7.73 d (3H, 2,4-diCl-C_6_H_3_, *J* = 8.6, 2.4 Hz); 8.16 s (1H, CH=N); 10.11 s, 12.08 s (2H, NHCSNH); 12.16 s (1H, NH, thiazolidine). ^13^C NMR (DMSO-d_6_) δ (ppm): 36.5; 46.9; 122.5; 127.8; 129.3; 129.4; 131.9; 132.0; 132.4; 132.7; 136.4; 142.7; 151.9; 169.4; 172.7; 175.9; 177.4. Anal. calc. for C_19_H_14_Cl_2_N_4_O_4_S_2_ (%): C 45.88; H 2.84; N 11.26. Found: C 45.67; H 2.80; N 11.23.

4-[{2-[(2,6-Dichlorophenyl)carbamothioyl]hydrazinylidene}methyl]phenyl (2,4-dioxo-1,3-thiazolidin-5-yl)acetate (**24**)

Yield 89%, mp 188–190 °C. ^1^H NMR (DMSO-d_6_) δ (ppm): 3.42–3.45 m (2H, CH-C**H**_2_); 4.86 dd (1H, C**H**-CH_2_, *J* = 6.9, 5.3 Hz); 7.21 d, 7.98 d (4H, 4-O-C_6_H_4_, *J* = 8.7 Hz); 7.38 dd, 7.56 d (3H, 2,6-diCl-C_6_H_3_, *J* = 8.6, 7.7 Hz); 8.15 s (1H, CH=N); 10.13 s, 12.03 s (2H, NHCSNH); 12.16 s (1H, NH, thiazolidine). ^13^C NMR (DMSO-d_6_) δ (ppm): 36.4; 46.8; 122.4; 128.8; 129.3; 129.9; 132.5; 135.6; 135.7; 142.3; 151.7; 169.4; 172.7; 175.9; 177.8. Anal. calc. for C_19_H_14_Cl_2_N_4_O_4_S_2_ (%): C 45.88; H 2.84; N 11.26. Found: C 45.89; H 2.77; N 11.18.

4-[{2-[(2-Chlorophenyl)carbamothioyl]hydrazinylidene}methyl]-2-methoxyphenyl (2,4-dioxo-1,3-thiazolidin-5-yl)acetate (**25**)

Yield 82%, mp 204–205 °C. ^1^H NMR (DMSO-d_6_) δ (ppm): 3.40–3.42 m (2H, CH-C**H**_2_); 3.83 s (3H, OCH_3_); 4.84 dd (1H, C**H**-CH_2_, *J* = 6.7, 5.5 Hz); 7.16 d, 7.56 dd, 7.66 d (3H, 4-O-C_6_H_3_, *J* = 7.8, 1.7 Hz); 7.29–7.43 m, 7.74 dd (4H, 2-Cl-C_6_H_4_, *J* = 7.8, 1.7 Hz); 8.14 s (1H, CH=N); 10.10 s, 12.06 s (2H, NHCSNH); 12.13 s (1H, NH, thiazolidine). ^13^C NMR (DMSO-d_6_) δ (ppm): 36.1; 46.9; 56.6; 111.0; 121.5; 123.4; 127.7; 128.4; 129.8; 130.4; 131.3; 133.6; 137.0; 140.9; 142.6; 151.5; 168.8; 172.7; 175.8; 177.1. Anal. calc. for C_20_H_17_ClN_4_O_5_S_2_ (%): C 48.73; H 3.48; N 11.37. Found: C 48.67; H 3.50; N 11.33.

4-[{2-[(3-Chlorophenyl)carbamothioyl]hydrazinylidene}methyl]-2-methoxyphenyl (2,4-dioxo-1,3-thiazolidin-5-yl)acetate (**26**)

Yield 62%, mp 156–158 °C. ^1^H NMR (DMSO-d_6_) δ (ppm): 3.40–3.42 m (2H, CH-C**H**_2_); 3.85 s (3H, OCH_3_); 4.84 dd (1H, C**H**-CH_2_, *J* = 6.6, 5.6 Hz); 7.17 d, 7.48 dd, 7.65 d (3H, 4-O-C_6_H_3_, *J* = 8.2, 1.8 Hz); 7.26–7.30 m, 7.40 t, 7.56–7.59 m, 7.76 t (4H, 3-Cl-C_6_H_4_, *J* = 8.0, 2.0 Hz); 8.15 s (1H, CH=N); 10.18 s, 12.03 s (2H, NHCSNH); 12.13 s (1H, NH, thiazolidine). ^13^C NMR (DMSO-d_6_) δ (ppm): 36.1; 47.0; 56.7; 111.7; 121.3; 123.4; 125.1; 125.7; 126.0; 130.1; 132.7; 133.6; 140.9; 141.0; 143.1; 151.4; 168.8; 172.8; 176.0; 176.4. Anal. calc. for C_20_H_17_ClN_4_O_5_S_2_ (%): C 48.73; H 3.48; N 11.37. Found: C 48.70; H 3.43; N 11.38.

4-[{2-[(4-Chlorophenyl)carbamothioyl]hydrazinylidene}methyl]-2-methoxyphenyl (2,4-dioxo-1,3-thiazolidin-5-yl)acetate (**27**)

Yield 90%, mp 200-203°C. ^1^H NMR (DMSO-d_6_) δ (ppm): 3.40–3.42 m (2H, CH-C**H**_2_); 3.85 s (3H, OCH_3_); 4.84 dd (1H, C**H**-CH_2_, *J* = 6.6, 5.5 Hz); 7.17 d, 7.42–7.49 m, 7.60–7.66 m (7H, 4-O-C_6_H_3_ & 4-Cl-C_6_H_4_, *J* = 8.2 Hz); 8.15 s (1H, CH=N); 10.16 s, 11.98 s (2H, NHCSNH); 12.14 s (1H, NH, thiazolidine). ^13^C NMR (DMSO-d_6_) δ (ppm): 36.1; 46.9; 56.7; 111.6; 121.3; 123.4; 128.3; 128.5; 130.0; 133.6; 138.5; 140.9; 142.9; 151.4; 168.8; 172.7; 175.8; 176.6. Anal. calc. for C_20_H_17_ClN_4_O_5_S_2_ (%): C 48.73; H 3.48; N 11.37. Found: C 48.62; H 3.44; N 11.29.

4-[{2-[(2,4-Dichlorophenyl)carbamothioyl]hydrazinylidene}methyl]-2-methoxyphenyl (2,4-dioxo-1,3-thiazolidin-5-yl)acetate (**28**)

Yield 92%, mp 196–198 °C. ^1^H NMR (DMSO-d_6_) δ (ppm): 3.40–3.43 m (2H, CH-C**H**_2_); 3.84 s (3H, OCH_3_); 4.84 dd (1H, C**H**-CH_2_, *J* = 6.6, 5.4 Hz); 7.17 d, 7.43 dd, 7.64 d (3H, 4-O-C_6_H_3_, *J* = 8.2, 1.8 Hz); 7.48 dd, 7.72–7.75 m (3H, 2,4-diCl-C_6_H_3_, *J* = 8.6, 2.4 Hz); 8.14 s (1H, CH=N); 10.08 s (1H, NHCSNH); 12.13 s (2H, NHCSNH and NH thiazolidine). ^13^C NMR (DMSO-d_6_) δ (ppm): 36.1; 46.9; 56.7; 111.2; 121.4; 123.4; 127.9; 129.3; 131.8; 131.9; 132.6; 133.6; 136.3; 140.9; 142.9; 151.5; 168.8; 172.7; 175.8; 177.2. Anal. calc. for C_20_H_16_Cl_2_N_4_O_5_S_2_ (%): C 45.55; H 3.06; N 10.62. Found: C 45.67; H 3.08; N 10.59.

4-[{2-[(2,6-Dichlorophenyl)carbamothioyl]hydrazinylidene}methyl]-2-methoxyphenyl (2,4-dioxo-1,3-thiazolidin-5-yl)acetate (**29**)

Yield 92%, mp 206–207 °C. ^1^H NMR (DMSO-d_6_) δ (ppm): 3.41 d (2H, CH-C**H**_2_, *J* = 6.3 Hz); 3.84 s (3H, OCH_3_); 4.84 t (1H, C**H**-CH_2_, *J* = 6.3 Hz); 7.17 d, 7.45 dd, 7.65 d (3H, 4-O-C_6_H_3_, *J* = 8.2, 1.6 Hz), 7.39 dd, 7.57 d (3H, 2,6-diCl-C_6_H_3_, *J* = 8.7, 7.6 Hz); 8.13 s (1H, CH=N); 10.06 s, 12.07 s (2H, NHCSNH); 12.13 s (1H, NH, thiazolidine). ^13^C NMR (DMSO-d_6_) δ (ppm): 36.1; 46.9; 56.8; 111.4; 121.4; 123.4; 128.9; 130.0; 133.7; 135.6; 135.8; 140.8; 142.8; 151.5; 168.8; 172.8; 175.9; 177.7. Anal. calc. for C_20_H_16_Cl_2_N_4_O_5_S_2_ (%): C 45.55; H 3.06; N 10.62. Found: C 45.57; H 3.03; N 10.61.

2-[{2-[(2-Chlorophenyl)carbamothioyl]hydrazinylidene}methyl]phenyl (2,4-dioxo-1,3-thiazolidin-5-ylidene)acetate (**30**)

Yield 81%, mp 222–223 °C. ^1^H NMR (DMSO-d_6_) δ (ppm): 7.14 s (1H, CH=); 7.29–7.42 m, 7.50–7.63 m, 8.34 dd (8H, 2-O-C_6_H_4_ and 2-Cl-C_6_H_4_, *J* = 7.8, 1.5 Hz); 8.30 s (CH=N); 10.06 s, 11.89 s (2H, NHCSNH); 13.02 s (1H, NH, thiazolidine). ^13^C NMR (DMSO-d_6_) δ (ppm): 116.3; 123.2; 126.5; 127.3; 127.4; 127.7; 128.6; 129.8; 130.7; 131.5; 131.7; 136.9; 137.9; 145.8; 149.2; 164.3; 166.3; 169.2; 177.3. Anal. calc. for C_19_H_13_ClN_4_O_4_S_2_ (%): C 49.51; H 2.84; N 12.16. Found: C 49.56; H 2.87; N 12.11.

2-[{2-[(3-Chlorophenyl)carbamothioyl]hydrazinylidene}methyl]phenyl (2,4-dioxo-1,3-thiazolidin-5-ylidene)acetate (**31**)

Yield 85%, mp 220–222 °C. ^1^H NMR (DMSO-d_6_) δ (ppm): 7.15 s (1H, CH=); 7.25–7.44 m, 7.50–7.61 m, 7.71–7.75 m, 8.38 dd (8H, 2-O-C_6_H_4_ and 3-Cl-C_6_H_4_, *J* = 7.8, 1.4 Hz); 8.31 s (CH=N); 10.18 s, 11.85 s (2H, NHCSNH); 13.04 s (1H, NH, thiazolidine). ^13^C NMR (DMSO-d_6_) δ (ppm): 116.3; 123.2; 124.7; 125.6; 125.7; 126.4; 127.3; 127.6; 130.1; 131.8; 132.7; 138.2; 140.9; 145.8; 149.3; 164.3; 166.4; 169.2; 176.4. Anal. calc. for C_19_H_13_ClN_4_O_4_S_2_ (%): C 49.51; H 2.84; N 12.16. Found: C 49.36; H 2.67; N 12.13.

3-[{2-[(2-Chlorophenyl)carbamothioyl]hydrazinylidene}methyl]phenyl (2,4-dioxo-1,3-thiazolidin-5-ylidene)acetate (**32**)

Yield 93%, mp 222–224 °C. ^1^H NMR (DMSO-d_6_) δ (ppm): 7.05 s (1H, CH=); 7.27–7.41 m, 7.49–7.61 m, 7.71 d, 7.91–7.95 m (8H, 3-O-C_6_H_4_ and 2-Cl-C_6_H_4_, *J* = 7.8 Hz); 8.17 s (1H, CH=N); 10.16 s, 12.06 s (2H, NHCSNH); 12.91 s (1H, NH, thiazolidine). ^13^C NMR (DMSO-d_6_) δ (ppm): 116.5; 119.9; 123.6; 127.7; 128.6; 129.8; 130.5; 131.0; 131.8; 136.4; 137.1; 142.0; 145.5; 150.8; 162.8; 164.4; 166.4; 169.4; 177.6. Anal. calc. for C_19_H_13_ClN_4_O_4_S_2_ (%): C 49.51; H 2.84; N 12.16. Found: C 49.53; H 2.81; N 12.07.

4-[{2-[(2-Chlorophenyl)carbamothioyl]hydrazinylidene}methyl]phenyl (2,4-dioxo-1,3-thiazolidin-5-ylidene)acetate (**33**)

Yield 83%, mp 224–226 °C. ^1^H NMR (DMSO-d_6_) δ (ppm): 7.07 s (1H, CH=); 7.31–7.42 m, 7.56 dd, 7.67 dd (6H, 4-O-C_6_H_4_ and 2-Cl-C_6_H_4_, *J* = 7.8, 1.5 Hz); 7.99 d (2H, 4-O-C_6_H_4_, *J* = 8.8 Hz) 8.18 s (CH=N); 10.15 s, 12.04 s (2H, NHCSNH); 12.93 s (1H, NH, thiazolidine). ^13^C NMR (DMSO-d_6_) δ (ppm): 116.7; 122.4; 127.7; 128.4; 129.3; 129.8; 130.6; 131.4; 132.8; 137.1; 142.3; 145.3; 151.6; 164.4; 166.5; 169.4; 177.3. Anal. calc. for C_19_H_13_ClN_4_O_4_S_2_ (%): C 49.51; H 2.84; N 12.16. Found: C 49.45; H 2.77; N 12.14.

4-[{2-[(3-Chlorophenyl)carbamothioyl]hydrazinylidene}methyl]phenyl (2,4-dioxo-1,3-thiazolidin-5-ylidene)acetate (**34**)

Yield 84%, mp 214–216 °C. ^1^H NMR (DMSO-d_6_) δ (ppm): 7.07 s (1H, CH=); 7.25–7.43 m, 7.57–7.60 m, 7.73–7.77 m, 8.01 d (8H, 3-Cl-C_6_H_4_ and 4-O-C_6_H_4_, *J* = 8.7 Hz); 8.18 s (1H, CH=N); 10.22 s, 12.00 s (2H, NHCSNH); 12.93 s (1H, NH, thiazolidine). ^13^C NMR (DMSO-d_6_) δ (ppm): 116.8; 122.4; 124.8; 125.5; 125.8; 129.5; 130.1; 132.6; 132.7; 141.0; 142.7; 145.3; 151.7; 164.2; 166.4; 169.4; 176.4. Anal. calc. for C_19_H_13_ClN_4_O_4_S_2_ (%): C 49.51; H 2.84; N 12.16. Found: C 49.47; H 2.74; N 12.04.

4-[{2-[(4-Chlorophenyl)carbamothioyl]hydrazinylidene}methyl]phenyl (2,4-dioxo-1,3-thiazolidin-5-ylidene)acetate (**35**)

Yield 86%, mp 214–216 °C. ^1^H NMR (DMSO-d_6_) δ (ppm): 7.06 s (1H, CH=); 7.32 d, 8.01 d (4H, 4-O-C_6_H_4_, *J* = 8.7 Hz); 7.43 d, 7.61 d (4H, 4-Cl-C_6_H_4_, *J* = 8.7 Hz); 8.18 s (CH=N); 10.20 s, 11.97 s (2H, NHCSNH); 12.97 s (1H, NH, thiazolidine). ^13^C NMR (DMSO-d_6_) δ (ppm): 116.8; 122.3; 128.1; 128.4; 129.5; 129.9; 132.7; 138.5; 142.5; 145.3; 151.6; 164.2; 166.4; 169.4; 176.5. Anal. calc. for C_19_H_13_ClN_4_O_4_S_2_ (%): C 49.51; H 2.84; N 12.16. Found: C 49.41; H 2.64; N 12.02.

4-[{2-[(2,4-Dichlorophenyl)carbamothioyl]hydrazinylidene}methyl]phenyl (2,4-dioxo-1,3-thiazolidin-5-ylidene)acetate (**36**)

Yield 79%, mp 230–232 °C. ^1^H NMR (DMSO-d_6_) δ (ppm): 7.07 s (1H, CH=); 7.32 d, 7.98 d (4H, 4-O-C_6_H_4_, *J* = 8.7 Hz); 7.47 dd, 7.64-7.70 m, 7.73 d (3H, 2,4-diCl-C_6_H_3_, *J* = 8.6, 2.4 Hz); 8.17 s (CH=N); 10.13 s, 12.09 s (2H, NHCSNH); 12.98 s (1H, NH, thiazolidine). ^13^C NMR (DMSO-d_6_) δ (ppm): 116.8; 122.4; 127.8; 129.3; 129.4; 131.9; 132.0; 132.7; 132.8; 136.4; 142.6; 145.3; 151.6; 164.2; 166.4; 169.4; 177.4. Anal. calc. for C_19_H_12_Cl_2_N_4_O_4_S_2_ (%): C 46.07; H 2.44; N 11.31. Found: C 46.09; H 2.44; N 11.22.

4-[{2-[(2-Chlorophenyl)carbamothioyl]hydrazinylidene}methyl]-2-methoxyphenyl (2,4-dioxo-1,3-thiazolidin-5-ylidene)acetate (**37**)

Yield 84%, mp 212–214 °C. ^1^H NMR (DMSO-d_6_) δ (ppm): 3.84 s (3H, OCH_3_); 7.08 s (1H, CH=); 7.27 d, 7.56 dd, 7.69 d (3H, 4-O-C_6_H_3_, *J* = 8.1, 1.7 Hz); 7.32–7.47 m, 7.71–7.78 m (4H, 2-Cl-C_6_H_4_); 8.16 s (CH=N); 10.11 s, 12.07 s (2H, NHCSNH); 12.93 s (1H, NH, thiazolidine). ^13^C NMR (DMSO-d_6_) δ (ppm): 56.7; 111.1; 116.0; 121.5; 123.5; 127.7; 128.4; 129.8; 130.4; 131.4; 134.0; 137.0; 140.5; 142.6; 146.0; 151.4; 163.7; 166.4; 169.3; 177.2. Anal. calc. for C_20_H_15_ClN_4_O_5_S_2_ (%): C 48.93; H 3.08; N 11.41. Found: C 46.09; H 2.44; N 11.22. 

4-[{2-[(3-Chlorophenyl)carbamothioyl]hydrazinylidene}methyl]-2-methoxyphenyl (2,4-dioxo-1,3-thiazolidin-5-ylidene)acetate (**38**)

Yield 80%, mp 190–192 °C. ^1^H NMR (DMSO-d_6_) δ (ppm): 3.86 s (3H, OCH_3_); 7.08 s (1H, CH=); 7.28 d, 7.40 t, 7.52 dd, 7.55–7.61 m, 7.69 d, 7.75–7.79 m (7H, 4-O-C_6_H_3_ and 3-Cl-C_6_H_4_, *J* = 8.1, 1.4 Hz); 8.17 s (1H, CH=N); 10.20 s, 12.04 s (2H, NHCSNH); 12.96 s (1H, NH, thiazolidine). ^13^C NMR (DMSO-d_6_) δ (ppm): 56.7; 111.8; 116.0; 121.3; 123.4; 125.1; 125.7; 126.0; 130.1; 132.7; 133.9; 140.5; 141.0; 143.0; 145.9; 151.3; 163.7; 166.3; 169.3; 176.4. Anal. calc. for C_20_H_15_ClN_4_O_5_S_2_ (%): C 48.93; H 3.08; N 11.41. Found: C 48.89; H 3.10; N 11.37.

4-[{2-[(4-Chlorophenyl)carbamothioyl]hydrazinylidene}methyl]-2-methoxyphenyl (2,4-dioxo-1,3-thiazolidin-5-ylidene)acetate (**39**)

Yield 82%, mp 196–198 °C. ^1^H NMR (DMSO-d_6_) δ (ppm): 3.87 s (3H, OCH_3_); 7.08 s (1H, CH=); 7.27 d, 7.53 dd, 7.69 d, (3H, 4-O-C_6_H_3_, *J* = 8.1, 1.8 Hz); 7.42 d, 7.60 d (4H, 4-Cl-C_6_H_4_, *J* = 8.7 Hz); 8.16 s (CH=N); 10.20 s, 12.05 s (2H, NHCSNH); 12.94 s (1H, NH, thiazolidine). ^13^C NMR (DMSO-d_6_) δ (ppm): 56.7; 111.4; 116.0; 121.4; 123.5; 128.4; 128.6; 130.0; 133.6; 138.4; 140.8; 142.9; 145.8; 151.4; 163.6; 166.4; 169.4; 176.5. Anal. calc. for C_20_H_15_ClN_4_O_5_S_2_ (%): C 48.93; H 3.08; N 11.41. Found: C 48.90; H 3.05; N 11.38.

4-[{2-[(2,4-Dichlorophenyl)carbamothioyl]hydrazinylidene}methyl]-2-methoxyphenyl (2,4-dioxo-1,3-thiazolidin-5-ylidene)acetate (**40**)

Yield 79%, mp 202–204 °C. ^1^H NMR (DMSO-d_6_) δ (ppm): 3.85 s (3H, OCH_3_); 7.09 s (1H, CH=); 7.46 dd, 7.62–7.67 m, 7.73 d (3H, 2,4-diCl-C_6_H_3_, *J* = 8.6, 2.4 Hz); 7.28 d, 7.53 dd, 7.69 d, (3H, 4-O-C_6_H_3_, *J* = 8.1, 1.8 Hz); 8.18 s (CH=N); 10.21 s, 12.03 s (2H, NHCSNH); 12.95 s (1H, NH, thiazolidine). ^13^C NMR (DMSO-d_6_) δ (ppm): 56.6; 111.2; 115.9; 121.5; 123.5; 127.9; 129.3; 131.7; 131.8; 132.6; 133.6; 136.4; 140.9; 142.9; 146.0; 151.3; 163.7; 166.5; 169.5; 176.6. Anal. calc. for C_20_H_14_Cl_2_N_4_O_5_S_2_ (%): C 45.72; H 2.69; N 10.66. Found: C 45.57; H 2.59; N 10.63.

#### 3.1.2. Synthesis of 4-[(2,5-dioxoimidazolidin-4-ylidene)methyl]-2-methoxyphenyl (2,4-dioxo-1,3-thiazolidin-5-yl/ylidene)acetates (**41**, **42**)

The solution of 0.01 mol acid chloride (**3** or **4**) in 3 mL of anhydrous dioxane was added to a solution of 0.01 mol of 5-(4-hydroxy-3-methoxybenzylidene)-imidazolidine-2,4-dione in 5 mL anhydrous pyridine. After 2 h, water was added and the mixture was acidified of diluted hydrochloric acid solution to pH = 3–4 and left at room temperature for 24 h. The precipitate was filtered off and then crystallized from n-butanol.

4-[(2,5-Dioxoimidazolidin-4-ylidene)methyl]-2-methoxyphenyl (2,4-dioxo-1,3-thiazolidin-5-ylidene)acetate (**41**)

Yield 71%, mp 187–189 °C. ^1^H NMR (DMSO-d_6_) δ (ppm): 3.85 s (3H, OCH_3_); 6.44 s (1H, CH=); 7.07 s (1H, =CH-COO); 7.20–7.31 m (3H, 4-O-C_6_H_3_); 10.67 s, 11.29 s (2H, 2NH, hydantoin); 12.95 bs (1H, NH, thiazolidine). ^13^C NMR (DMSO-d_6_) δ (ppm): 56.6; 108.1; 114.1; 116.0; 122.6; 123.4; 128.7; 133.1; 139.0; 145.8; 151.1; 156.3; 163.7; 166.0; 166.3; 169.3.

4-[(2,5-Dioxoimidazolidin-4-ylidene)methyl]-2-methoxyphenyl (2,4-dioxo-1,3-thiazolidin-5-yl)acetate (**42**)

Yield 72%, mp 192–194 °C. ^1^H NMR (DMSO-d_6_) δ (ppm): 3.40–3.42 m (2H, CH-C**H_2_**); 3.84 s (3H, OCH_3_); 4.85 dd (1H, C**H**-CH_2_, *J* = 6.7, 5.5 Hz); 6.42 s (1H, CH=); 7.08 d, 7.21–7.28 m (3H, 4-O-C_6_H_3_, *J* = 8.4 Hz); 10.65 s, 11.28 s (2H, 2NH, hydantoin); 12.13 s (1H, NH, thiazolidine). ^13^C NMR (DMSO-d_6_) δ (ppm): 36.1; 46.9; 56.3; 110.2; 113.7; 116.2; 123.9; 124.8; 125.9; 148.2; 156.2; 164.4; 166.1; 168.9; 172.6; 175.8.

2-Ethoxy-4-[(5-oxo-2-thioxoimidazolidin-4-ylidene)methyl]phenyl (2,4-dioxo-1,3-thiazolidin-5-ylidene)acetate (**43**) 

was obtained by the method reported previously [21]. Spectral characteristics were presented in above mentioned publication.

### 3.2. Microbiology Tests

Twelve reference strains of bacteria from ATCC (American Type Culture Collection) were used. Among Gram-positive bacteria there were: *Staphylococcus aureus* ATCC 25923, *Staphylococcus aureus* ATCC 6538, S*taphylococcus epidermidis* ATCC 12228, *Bacillus subtilis* ATCC 6633, *Bacillus cereus* ATCC 10876, and *Micrococcus luteus* ATCC 10240. However, within Gram-negative bacteria there were species with low growth and atmosphere requirements from *Enterobacteriaceae* family (*Escherichia coli* ATCC 25922, *Klebsiella pneumoniae* ATCC 13883, *Proteus mirabilis* ATCC 12453) and from *Pseudomonadaceae* family (*Pseudomonas aeruginosa* ATCC 9027), as well as fastidious bacteria from *Pasteurellaceae* family (*Haemophilus influenzae* ATCC 10211 and *Haemophilus parainfluenzae* ATCC 51505) with high growth and atmosphere requirements. Microbial suspensions with an optical density of 150 × 10^6^ CFU/mL (CFU—colony forming units, 0.5 McFarland standard) were prepared in sterile 0.85% NaCl. 

The compounds’ stock solutions were dissolved in dimethyl sulfoxide (DMSO) with no inhibitory concentration on the growth of bacteria (negative control). The medium with DMSO without the tested compounds was used as negative control. A ciprofloxacin, cefuroxime and oxacillin were used as reference antimicrobials. 

The antibacterial activity of tested compounds was firstly screened by the agar dilution method on the basis of the microbial growth inhibition zone (giz, in mm, with 1000 mg/L concentration of the tested compounds). Then it was determined by broth microdilution technique with series of twofold dilution (final concentrations from 0.007 to 1000 mg/L), of the tested compounds or ciprofloxacin, cefuroxime and oxacillin as was described earlier [26]. The antimicrobial effect was detected with the Mueller–Hinton medium. 

The activity was expressed as the minimal inhibitory concentration (MIC) assayed spectrophotometrically by optical density determination (OD_600_). The MBC (minimal bactericidal concentration)—defined as the lowest concentration of each compound that resulted in >99.9% reduction in CFU of the initial inoculum—was also determined by plating out the 5 μL contents of wells with no visible growth of bacteria, onto Mueller–Hinton agar plates (incubation conditions 35 °C for 18 h). The compounds were classified as bacteriostatic when the MBC/MIC ratio was ≥8 and bactericidal when the MBC/MIC ratio was ≤4 [27].

### 3.3. Computational Details

Conformational search, physicochemical parameters, and HOMO/LUMO maps were calculated using HyperChem 8.0.1 [28]. Extensive conformational searches were carried out using the molecular mechanics level with OPLS force field [29]. The most stable structures obtained were subsequently optimized to the closest local minimum at the semiempirical level using RM1 parametrization. Convergence criteria were set to 0.1 and 0.01 kcal mol^−1^ Å^−1^ for OPLS and RM1 calculations, respectively. Electrostatic potentials were calculated using Gaussian 03 and GaussView 5 at the HF/6–31 G level.

### 3.4. Cytotoxicity Studies

Cytotoxic properties of the investigated compounds were evaluated using human embryonic kidney (HEK-293) cell line. HEK-293 cells were purchased from the American Type Culture Collection (ATCC CRL-1573) and were cultured using DMEM (Sigma–Aldrich) supplemented with 10% fetal bovine serum (FBS, Sigma–Aldrich, St. Louis, MO, USA), 100 U/mL of penicillin and 100 mg/L of streptomycin (PenStrep, Sigma–Aldrich). Cells were incubated at 37 °C in a humidified atmosphere of 5% CO_2_. Stock solutions of the tested compounds were prepared dissolving in a sterile dimethyl sulfoxide (DMSO) to the concentration of 100 mg/L. On the day of experiment, HEK-293 cells were seeded into 96-well sterile plates (Nunc) at a cell density of 3 × 10^5^ cells/well. After 24 h of incubation, the medium was removed from each well and then cells were incubated for the next 24 h with different concentrations of the tested compounds (1–250 mg/L) in DMEM containing 2% FBS. Control cells were cultured only with medium containing 2% addition of FBS. Toxicity of the compounds was evaluated using MTT assay, which is based on the conversion of 3-(4,5-dimethylthiazol-2-yl)-2,5-diphenyltetrazolium bromide (MTT) into dark-blue formazan crystals. Briefly, after 24 h incubation of cells with varying concentrations of tested compounds, all culture media were removed from the plates. Cells were washed with PBS, and then 100 µL of medium containing 10% MTT solution (5 mg/L) was added to each well. Cells were incubated for the next 4 h at 37 °C in an atmosphere of 5% CO_2_. Afterwards, 100 µL (per well) of 10% SDS buffer solution was added to dissolve formazan crystals and after an overnight incubation the absorbance was measured at 540 and 620 nm using a microplate reader (Epoch, BioTek Instruments, Luzern, Switzerland). The obtained results were expressed as EC_70_ ± SD from three independent experiments done in triplicate. EC_70_ represents the concentration of the compound that inhibited viability of cells by 30%.

## 4. Conclusions

The studied new thiazolidine-2,4-dione-chlorophenylthiosemicarbazone hybrids showed promising activity against Gram-positive bacterial strains. The most potent derivatives (**20**, **23**, **30**, **31** and **38**) exhibited good activity against all six Gram-positive strains used in microbiological studies. The compound **38** showed more effective action than standard drug cefuroxime against *B. subtilis* and *B. cereus*. These compounds were non-toxic in concentration close to their antibacterial effect. Moreover, the SARs analysis collectively suggests that at least two different molecular mechanisms of antibacterial activity for TZD-based hybrid compounds should be expected.

## Figures and Tables

**Table 1:** The activity of chlorophenylthiosemicarbazones (**17**–**40**) against Gram-positive bacteria determined on the basis of MIC (minimal inhibitory concentration, in mg/L and µM) and MBC (minimal bactericidal concentration, in mg/L and µM).

Compound	*S. aureus*		*S. aureus*		*S. epidermidis*		*B. subtilis*		*B. cereus*		*M. luteus*	
	ATCC 6538		ATCC 25923		ATCC 12228		ATCC 6633		ATCC 10876		ATCC 10240	
	MIC	MBC	MIC	MBC	MIC	MBC	MIC	MBC	MIC	MBC	MIC	MBC
**19**	125	>1000	62.5	>1000	62.5	>1000	62.5	500	62.5	>1000	62.5	1000
	251.3	>2010.6	125.7	>2010.6	125.7	>2010.6	125.7	1005.3	125.7	>2010.6	125.7	2010.6
**20**	3.91	3.91	3.91	62.5	31.25	62.5	31.25	125	3.91	125	7.81	125
	8.4	8.4	8.4	135	67.5	135	67.5	270	8.4	270	16.9	270
**21**	31.25	>1000	7.81	1000	31.25	>1000	62.5	250	31.25	250	15.63	15.63
	67.5	>2160.2	16.9	2160.2	67.5	>2160.2	135	540	67.5	540	33.8	33.8
**22**	15.63	1000	31.25	1000	15.63	1000	31.25	>1000	7.81	>1000	3.91	500
	33.8	2160.2	67.5	2160.2	33.8	2160.2	67.5	>2160.2	16.9	>2160.2	8.4	1080.1
**23**	3.91	>1000	3.91	>1000	7.81	1000	7.81	62.5	7.81	>1000	7.81	7.81
	7.9	>2010.6	7.9	>2010.6	15.7	2010.6	15.7	125.7	15.7	>2010.6	15.7	15.7
**24**	125	>1000	62.5	>1000	31.25	>1000	125	250	62.5	>1000	62.5	>1000
	251.3	>2010.6	125.7	>2010.6	62.8	>2010.6	251.3	502.6	125.7	>2010.6	125.7	>2010.6
**26**	15.63	>1000	15.63	>1000	31.25	>1000	15.63	500	62.5	>1000	3.91	1000
	31.7	>2028.6	31.7	>2028.6	63.4	>2028.6	31.7	1014.3	126.8	>2028.6	7.9	2028.6
**29**	125	>1000	62.5	>1000	31.25	>1000	31.25	500	15.63	1000	7.81	1000
	237	>1896.1	118.5	>1896.1	59.3	>1896.1	59.3	948	29.6	1896.1	14.8	1896.1
**30**	7.81	250	7.81	250	7.81	250	7.81	250	3.91	500	7.81	250
	16.9	542.4	16.9	542.4	16.9	542.4	16.9	542.4	8.5	1084.8	16.9	542.4
**31**	7.81	500	7.81	500	7.81	500	7.81	500	3.91	500	7.81	1000
	16.9	1084.8	16.9	1084.8	16.9	1084.8	16.9	1084.8	8.5	1084.8	16.9	2169.6
**33**	15.63	1000	15.63	250	31.25	500	15.63	500	62.5	>1000	62.5	>1000
	33.9	2169.6	33.9	542.4	67.8	1084.8	33.9	1084.8	135.6	>2169.6	135.6	>2169.6
**34**	31.25	500	7.81	500	15.63	500	15.63	500	7.81	1000	62.5	500
	67.8	1084.8	16.9	1084.8	33.9	1084.8	33.9	1084.8	16.9	2169.6	135.6	1084.8
**35**	500	1000	1000	500	500	500	500	500	1000	1000	1000	1000
	1084.8	2169.6	2169.6	1084.8	1084.8	1084.8	1084.8	1084.8	2169.6	2169.6	2169.6	2169.6
**36**	125	500	7.81	125	7.81	62.5	250	1000	7.81	1000	7.81	250
	252.3	1009.4	15.8	252.3	15.8	126.2	504.7	2018.7	15.8	2018.7	15.8	504.7
**38**	31.25	>1000	7.81	>1000	7.81	250	3.91	62.5	3.91	62.5	3.91	125
	63.7	>2036.9	15.9	>2036.9	15.9	509.2	8	127.3	8	127.3	8	254.6
**39**	250	1000	250	1000	500	1000	500	500	1000	1000	1000	1000
	509.2	2036.9	509.2	2036.9	1018.5	2036.9	1018.5	1018.5	2036.9	2036.9	2036.9	2036.9
**40**	500	1000	250	1000	250	1000	500	1000	1000	1000	1000	1000
	951.7	1903.4	475.8	1903.4	475.8	1903.4	951.7	1903.4	1903.4	1903.4	1903.4	1903.4
**Cefuroxime**	0.98	-	0.49	-	0.24	-	15.63	-	31.25	-	0.98	-
	2.3		1.2		0.6		36.8		73.6		2.3	
**Ciprofloxacin**	0.49	0.49	-	-	0.49	0.49	0.015	0.12	0.12	0.12	0.98	1.95
	1.5	1.5			1.5	1.5	0.05	0.4	0.4	0.4	3.0	5.9
**Oxacillin**	0.06	0.06	-	-	0.12	0.12	0.06	0.12	62.5	62.5	0.98	0.98
	0.15	0.15			0.3	0.3	0.15	0.3	155.7	155.7	2.4	2.4

- not determined, compounds **17**, **18**, **25**, **27**, **28**, **32** and **37**—not active.

**Table 2:** Cytotoxic effect of compounds **20**, **23**, **30**, **31** and **38** on human embryonic kidney cells (HEK-293) after 24 h of incubation.

Compound	EC_70_ ± SD (mg/L) (Toxicity Threshold)
**20**	30.82 ± 3.73
**23**	21.28 ± 3.84
**30**	14.86 ± 1.12
**31**	10.94 ± 1.35
**38**	19.01 ± 3.80
